# DNA methylation data from Japanese patients with Rubinstein–Taybi syndrome

**DOI:** 10.1038/s41439-025-00332-0

**Published:** 2025-11-28

**Authors:** Tomoko Kawai, Taiga Aoki, Kazuhiko Nakabayashi, Kenichiro Hata, Tadashi Kaname, Rika Kosaki

**Affiliations:** 1 Department of Maternal–Fetal Biology, National Center for Child Health and Development, Tokyo, Japan; 2 Department of Genome Medicine, National Center for Child Health and Development, Tokyo, Japan; 3 Department of Human Molecular Genetics, Gunma University Graduate School of Medicine, Gunma, Japan; 4 Division of Medical Genetics, National Center for Child Health and Development, Tokyo, Japan

**Keywords:** Diagnostic markers, Clinical epigenetics

## Abstract

An episignature is a genome-wide DNA methylation pattern that is specific to each syndrome or etiologic gene. Episignature analysis helps to diagnose patients with variants of uncertain significance (VUS), but this requires positive methylation datasets from patients with a definitive diagnosis. Here we provide methylation datasets of Rubinstein–Taybi syndrome at the individual patient level, which have not been published before. This dataset increases the likelihood of determining the function of the VUS.

Congenital neurodevelopmental disorders are often caused by pathogenic variants in genes encoding the epigenetic machinery, which consists of writers, erasers, readers and remodelers that play roles in the modification, demodification, recognition of DNA or histone proteins, and chromatin remodeling, respectively^1^. At the same time, pathogenic variants in the genes of the epigenetic machinery have been found to cause gene-specific genome-wide DNA methylation patterns, called episignatures, that are detectable in the peripheral blood of affected individuals^2^. Moreover, in some genes, episignatures differ between the loci of frameshift, nonsense or missense variants, examples of which include *CREBBP* and *EP300*. The CREB-binding protein (CBP), encoded by *CREBBP*, and its paralog E1A-associated protein (p300), encoded by *EP300*, are involved in histone acetylation and transcriptional regulation. Pathogenic variants in exons 30/31 of *CREBBP* or *EP300* are etiologic for Menke–Hennekam syndromes (MKHK) 1,2 (Online Mendelian Inheritance in Man (OMIM) nos. 618332 and 618333)^3^, whereas variants that produce a null allele or a functional change in the catalytic domain of either protein cause Rubinstein–Taybi syndrome (RSTS) 1,2 (OMIM nos. 180849 and 613684)^4^. RSTS is a multiple congenital anomaly syndrome characterized by intellectual disability, postnatal growth failure, microcephaly, broad thumbs and dysmorphic facial features. However, none of the patients with MKHK with developmental delay or intellectual disability have the broad or angulated thumbs or the characteristic facial features of RSTS^3–5^. The episignatures of RSTS and MKHK also differ, as do the phenotypes^2^, thereby indicating that these two syndromes differ at the cellular level as a consequence of epigenetic regulation^6^. The diagnostic guidelines for RSTS reported by an international consensus group have described molecular diagnostic pathways, including DNA methylation episignature analysis, for uncertain results of exome or genome sequencing in patients with nonclassical RSTS phenotypes^7^. Episignatures reveal the molecular dysregulation caused by congenital genetic disorders, which can aid in determining the pathogenicity of variants of uncertain significance (VUS)^8^. Pathogenicity determination for VUS using episignatures is performed using supervised classification models. One of the most widely used classifiers for differentiating cases and controls based on methylation data is support vector machines (SVM)^9^. Having identified support vectors using training sets consisting of episignatures from controls and cases with verified pathogenic variants, new cases with VUSs are classified using Platt-modified methods with a probability score between 0 and 1, indicating control and pathogenicity, respectively. Episignatures with scores above 0.50 or 0.75, which depend on the authors, for VUS are classified as pathogenic variants based on DNA methylation analysis^8,10–12^.

So far, there have been no reports of raw DNA methylation *β* values for the episignature probe sets of RSTSs in individual patients and, consequently, researchers are unable to construct support vectors for classifying individual participants with VUSs within *CREBBP* or *EP300*. Although previously published mean methylation *β* values of RSTS episignature probe sets obtained from 30 patients, together with mean *β* values for a further 33 syndromes^11^, can be utilized as positive controls for clustering analysis, this information is insufficient for SVM analysis. However, providing raw data, such as those for BAFopathy^10^, CHARGE^13^, Kabuki^13^, Sotos^14^, Williams^15^ and Wolf–Hirschhorn^8^ syndromes, increases the likelihood of diagnosing pathogenicity of VUS. In addition, DNA methylation is reversible in response to environmental factors and is influenced by nearby nucleotide sequences that affect DNA binding factors' accessibility. Moreover, sharing array data could provide opportunities to establish more robust episignature probe sets that are stable and independent of genetic background.

In this study, we examined the DNA methylation *β* values of the episignature probes for RSTSs (RSTS1 (*CREBBP*) and RSTS2 (*EP300*))^2,11^, of eight Japanese patients diagnosed with RSTS1 and one diagnosed with RSTS2 (Table 1). The experimental protocol was approved by the Committee for Ethical Issues at National Center for Child Health and Development (nos. 2020-326 and 2022-183). Written informed consent was obtained from the patients or guardians. All participants showed classical RSTS phenotypes: three harbor nonsense variants at the 5′ end of the catalytic domain, one harbors a small frameshift nonsense Delins variant, one harbors a single-nucleotide substitution at the splicing donor site, one harbors a small deletion at the exon–intron junction, one harbors a large deletion from exon 24 to the transcription termination site of *CREBBP* (NM_004380.3) and two harbor a large deletion from the neighboring gene to exon 2 of *CREBBP* (Fig. 1A). The final two of the aforementioned individuals are monozygotic twins (RSTS1_7, _8)^16^. DNA from peripheral blood cells was converted by bisulfite treatment and processed using Illumina Infinium EPIC bead chip arrays as previously described^8^. We used publicly available raw data files from the EPIC array, which were collected from blood cells of healthy children in various environments as a control group for congenital disorders. Very few files were obtainable (Supplementary Table 1). Our data were combined and co-processed with the above data. Briefly, methylated and unmethylated signal intensities were normalized using R version 4.3.1 and ENmix package version 1.38.1, with background and dye bias corrections. *β* Values were calculated by dividing methylated signals by (methylated + unmethylated) signals. *β* Values of the published episignature probe sets for RSTSs, RSTS1 and RSTS2 in the participants are presented in Supplementary Tables 2–4. Hierarchical clustering based on Manhattan distance using the average method distinguished the nine RSTS patients from controls corresponding to the *β* values of each of the RSTS and RSTS1 episignature probe sets. The RSTS2 probe set distinguished only the RSTS2_1 patient across a great distance (Fig. 1B)^2,11^. Multidimensional scaling based on scaling the pairwise Euclidean distances between samples revealed the common DNA methylation changes in the eight RSTS1 patients when focusing on the *β* values of the RSTS1 probe set, which was clearer than multidimensional scaling using the RSTS probe set. Furthermore, the *β* values for the RSTS2 probe set clearly classified the single RSTS2 patient from the RSTS1 patients and controls. These results reconfirmed that episignatures are specific for the *CREBBP* and *EP300* pathogenic variants. Furthermore, none of the control subjects without congenital disorders exhibited an RSTS episignature, even when exposed to various environmental and/or physical conditions.

**Fig. 1:**
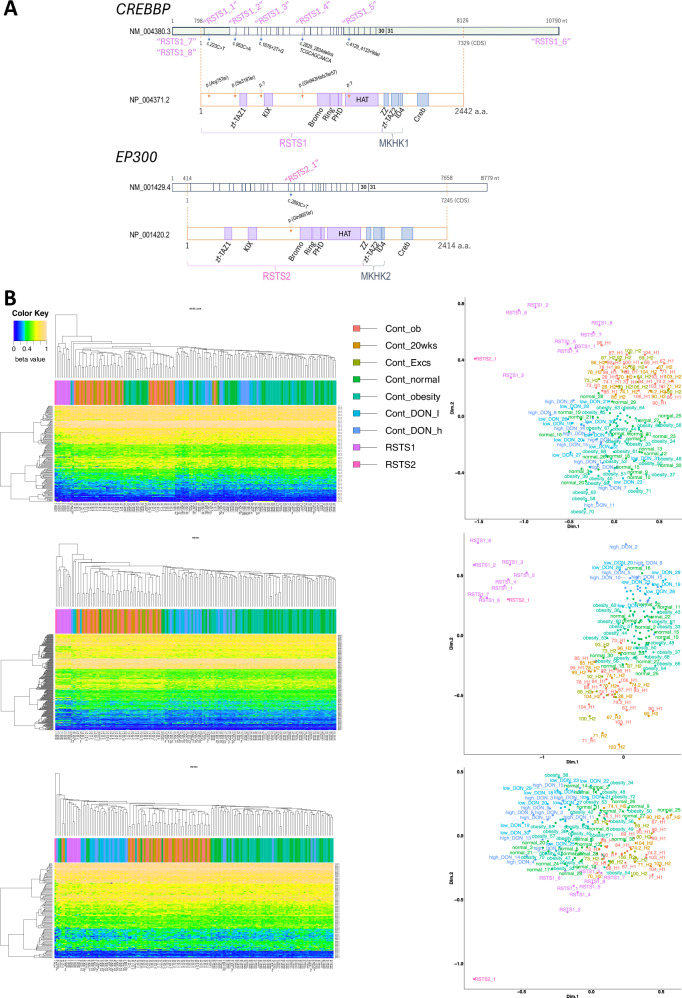
Classification of participants using episignatures. **A** The variants loci of participants in *CREBBP* or *EP300* in this study. **B** The participants (RSTS1 and RSTS2) were classified by DNA methylation of each probe set for RSTS episignatures using hierarchical clustering (left) or multidimensional scaling (right). Control data were obtained from publicly available data. Cont_ob, obese or overweight children in GSE193730; Cont_20wks and Cont_Excs, 20 wks after usual lifestyle and exercise intervention of Cont_ob, respectively, from GSE193730; Cont_normal and Cont_obesity, normal control and simple obesity children, respectively, from GSE221864; Cont_DON_l and Cont_DON_h, low and high exposure deoxynivalenol group children, respectively, from GSE180534.

**Table 1:** Pathogenic variants in the patients.

ID	Age (years)	Sex	Causative gene	DNA variant	Predicted protein consequence	Diagnostic scores (criteria for RSTS)	ACMG* classification	Diagnosis
RSTS1_1	4	Female	*CREBBP*	NM_004380.3:c.223C>T	p.(Arg75Ter)	9	Pathogenic	RSTS1
RSTS1_2	9	Female	*CREBBP*	NM_004380.3:c.953C>A	p.(Ser318Ter)	9	Pathogenic	RSTS1
RSTS1_3	3	Female	*CREBBP*	NM_004380.3:c.1676+2T>G	Splice donor variant, p.?	9	Likely Pathogenic	RSTS1
RSTS1_4	1	Male	*CREBBP*	NM_004380.3:c.2829_2834delins TCGCAGCAACA	p.(Gln943HisfsTer57)	10	Likely Pathogenic	RSTS1
RSTS1_5	14	Male	*CREBBP*	NM_004380.3:c.4129_4133+9del	Splicing junction loss, p.?	10	Likely Pathogenic	RSTS1
RSTS1_6	6	Female	*CREBBP*	arr[hg 19] 16p13.3 (3,739,149–3,791,328)× 1	Null	8	–	RSTS1
RSTS1_7	3	Male	*CREBBP*	arr[hg 18] 16p13.3 (3,810,524–4,233,361)× 1	Null	9	–	RSTS1
RSTS1_8	3	Male	*CREBBP*	arr[hg 18] 16p13.3 (3,810,524–4,233,361)× 1	Null	9	–	RSTS1
RSTS2_1	1	Male	*EP300*	NM_001429.4:c.2893C>T	p.(Gln965Ter)	9	Pathogenic	RSTS2

*ACMG* American College of Medical Genetics and Genomics.

Among our samples, RSTS1_3 harbors an intron variant in *CREBBP* (NM_004380.3:c.1676+2T>G). The corresponding locus is a well-established conserved nucleotide in the splicing donor site that influences mRNA splicing. Although this variant has not been previously reported in ClinVar^17^, in silico predictions, dbscSNV and MaxEntScan, identify this variant as a pathogenic variant^18,19^. Having removed RSTS1_3, training was performed using datasets from the remaining seven RSTS1 patients, and we obtained a RSTS1 probability of 0.985 in RSTS1_3 by test (Fig. 2A). The variant in RSTS1_3 was also classified as ‘pathogenic’ based on episignature analysis. High probability scores (>0.9) were also confirmed in the other seven RSTS1 patients by performing leave-one-out cross-validation (Fig. 2B). Meanwhile, we obtained a RSTS1 probability of 0.542 in RSTS2_1, which is lower than the border when 0.75 is adopted^8^. These results again showed that the episignatures between *CREBBP* and *EP300* pathogenic variants are indeed different. To examine whether the batch effects between the publicly available controls and our original data are involved in the results, we calculated the RSTS1 probability in a patient with Prader–Willi syndrome (PWS), which was assessed by the same batch as the nine RSTSs in this study, and previously reported 28 congenital disease cases^8^ (Fig. 2A). All disease cases showed RSTS1 probability scores of less than 0.3, which were much lower than the threshold of 0.75. The SVM model in this study worked accurately.

**Fig. 2:**
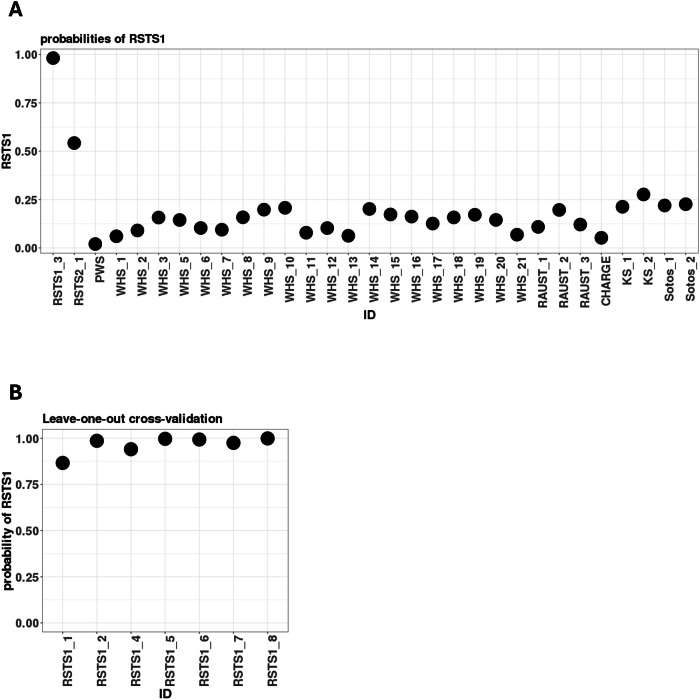
Probability scores of testing set. **A** The SVM was trained by the seven RSTS1 patients and publicly available 150 samples from control children. Testing set composed of RSTS1 with an intron variant (RSTS1_3), RSTS2, PWS and 28 congenital disease cases^8^ (20 Wolf–Hirschhorn syndromes (WHS), three Rauch–Steindl syndrome (RAUST), one CHARGE syndrome (CHARGE), two Kabuki syndrome (KS) and two Sotos syndrome (Sotos)) was calculated to determine the probability of RSTS1. **B** The probability of RSTS1 in each RSTS1 patient was calculated by leave-one-out cross-validation.

There are 60 and 34 intron variants in *CREBBP* and *EP300*, respectively, that are classified as either ‘Conflicting classifications of pathogenicity’ or ‘Uncertain significance’ in ClinVar^17^. The detection of intron variants with ‘uncertain significance’ will be further enhanced by whole-genome analysis. The *β* values of the episignatures published by this study could contribute to identifying the pathogenicity of these intron variants, as well as missense VUS. Furthermore, with respect to *CREBBP* and *EP300* in particular, the loci of the variants in which domain are essential for phenotypes and episignatures^3,6^. The data obtained in this study could provide an opportunity to apply episignature analysis in classifying the variants of RSTS or MKHK.

## HGV Database

The relevant data from this Data Report are hosted at the Human Genome Variation Database at 10.6084/m9.figshare.hgv.3560, 10.6084/m9.figshare.hgv.3563, 10.6084/m9.figshare.hgv.3566, 10.6084/m9.figshare.hgv.3569, 10.6084/m9.figshare.hgv.3572, 10.6084/m9.figshare.hgv.3575, 10.6084/m9.figshare.hgv.3578, 10.6084/m9.figshare.hgv.3581.

## Supplementary information

Supplementary Table 1

Supplementary Table 2

Supplementary Table 3

Supplementary Table 4
